# Fractal dimension in CBCT images as predictor for MRONJ: a retrospective cohort study

**DOI:** 10.1007/s00784-020-03523-x

**Published:** 2020-08-22

**Authors:** R. Bachtler, Ch. Walter, Ralf K. W. Schulze

**Affiliations:** 1 Department of Oral and Maxillofacial Surgery, Section of Oral Radiology, University Medical Center of the Johannes Gutenberg-University, Augustusplatz 2, 55131 Mainz, Germany; 2 MKG-Chirurgie, Medi+ Zahnärztliche Praxisklinik, Mainz, Germany

**Keywords:** CBCT, MRONJ, Fractal dimension

## Abstract

**Objective:**

To estimate boxcounting fractal dimension in a standardized approach in CBCT images of the mandible and maxilla in a group of patients with MRONJ in comparison to a healthy control group.

**Material and methods:**

From our records, a study group consisting of 80 maxillary and mandibular CBCTs in 77 cases of patients having MRONJ was collected. The control group consisted of 84 mandibular and maxillary CBCTs in a total of 78 patients. Using the boxcounting method, fractal dimension (FD) was estimated in a standardized fashion either cranially to the apex of the canine (maxilla) or beneath the tooth apices of the lower molars in the bone area above the mandibular canal (mandible). Intra-observer reproducibility of the FD-measurements was assessed by 6 repeated measurements in 10 individuals. FD values were correlated to age and sex of the individuals as well as to region of interest (ROI) sizes.

**Results:**

FD in the study group (1.684 ± 0.051) was roughly 3.5 % lower than in the control group (1.745 ± 0.026, *p* < 0.0001). Sex and age had a significant (*p* < 0.001) influence on FD values in the study group, yet not in the control group. FD values increased with age (study group, spearman-rho: 0.2895, *p* < 0.05) and also ROI size (both groups, *p* < 0.0001). Reproducibility was good (intra-class correlation coefficient (ICC): 0.87).

**Conclusions:**

Fractal dimension as assessed by boxcounting seems to be a good descriptor for MRONJ in jaw bones. Influence of age and sex on the outcome values needs to be further investigated in future studies.

**Clinical relevance:**

CBCTs could be assessed with respect to FD to obtain an overview of the disease status of MRONJ patients.

## Introduction

Medication-related osteonecrosis of the jaw (MRONJ) is the common term for a group of diseases that are side effects of antiresorptive and antiangiogenic agents. According to the position paper of the American Association of Oral and Maxillofacial Surgeons [1], MRONJ is considered to be present in patients with the following criteria [1]:

1. Current or previous treatment with antiresorptive or antiangiogenic agents
2. Plus have no history of radiation therapy to the jaws or obvious metastatic disease to the jaw
3. Plus who exhibit probably exposed bone of the jaws for a minimum period of 8 weeks

The main share of the cases is caused by antiresorptive drugs (including bisphosphonates and denosumab) [2], with prevalence rates in patients treated for malignancies as high as 21% [3]. Early-stage identification of MRONJ patients is thus essential. Clinical findings tend to underestimate the extent of the disease [4]. Thus, most commonly, three-dimensional radiographic imaging is proposed for diagnosis [5, 6]. Typical radiographic signs of MRONJ include osteosclerosis of the jaw bones, osteolysis, dense woven bone, a thickened lamina dura, subperiosteal bone deposition, and persisting alveolar sockets after tooth extraction with lacking bone remodeling [6].

Fractal dimension (FD) describes a ratio (index) of complexity comparing how detail of a pattern changes with the scale it is measured. The concept of a non-integer (fractional) or fractal dimension had originally been introduced by Mandelbrot in 1967 [7]. It has been applied to various areas in science since it provides a measure for complexity and irregularity of a given object [8]. Here, complexity is change in detail with change in scale. Higher values for the fractal dimension correlate with higher complexity (“roughness”) of the surface of an object [8]. The concept of using FD as a metric in radiographic analysis has been successfully applied in mammography [9–11] and also for describing trabecular patterns in bone [12, 13]. We are also aware of one article using it for MRONJ assessment in computed tomography (CT) images [14]. As sclerosis reduces the typical (regular) trabecular pattern in bones, one would expect that FD is reduced in MRONJ patients when compared with healthy controls.

The aim of this study was to estimate fractal dimension in a standardized approach in CBCT images of the mandible and maxilla in a group of patients with MRONJ as defined above versus a similar group of healthy patients. Null hypothesis was that FD does not differ between these two groups.

## Methods

### Patient groups

From the existing records of one author (CW), 45 MRONJ patients (only bisphosphonate medication, no other antiresorptive drugs) were identified that also had a CBCT (all 3D Accuitomo 80, J Morita Corp., Kyoto, Japan) including parts of their mandibular body. The same was done for 35 patients with existing CBCTs of their maxillary. All patients had clinically visible osteonecrosis and thus were stage 3 patients. This was the study group all presenting a region of interest (ROI, defined below) to be available in their CBCT scan at least for one patient side. The records of this study group included all relevant medical information, e. g., basic illness, medication, medication time, and patient history. Maxillary and mandibular CBCTs were from the same patients in 3 cases; all other data were from different individuals. It is important to note that the ROI was selected according to anatomical criteria as specified below regardless of the fact if the bone in this region was necrotic or not.

In an effort to match a group of healthy individuals, from our database of CBCT images by manual search, a group (according to their digital records also accessible in our University Medical Center) of healthy subjects were identified also having a CBCT scan available (control group: 42 andibular and maxillary CBCTs with an overlap of 6 patients which were in both groups). Patients with known bone diseases (such as osteoporosis or osteopenia) or osteo-modulating drugs were excluded from the study. As most CBCTs available in our database from healthy patients are from younger patients, unfortunately this resulted in a significantly lower age distribution in the control group.

### Image assessment

CBCT images were all assessed in their native voxel-size (0.08 mm). As described above, ROI selection was driven by anatomical criteria only. For mandibular assessments, square ROIs were sampled similarly to Torres et al. [14] beneath the tooth apices of the lower molars in the bone area above the mandibular canal. In the maxilla, ROIs were positioned cranially of the root of the canine. The size of the ROIs (range between 43 × 43 pixels and 100 × 100 pixels) differed as it was determined by the maximal area at that location only covering spongious bone.

**Fig. 1:**
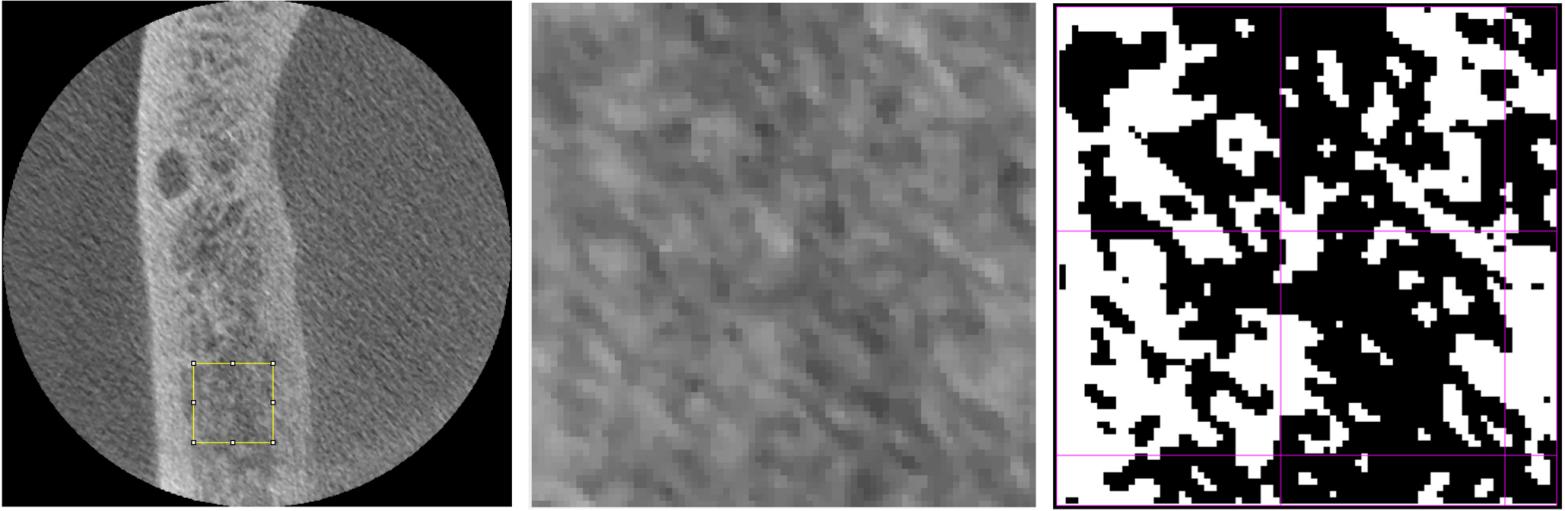
The ROI (middle image) placed in the axial slice (left image) is subsequently by the FracLac tool translated into a binary (black/white) image for further processing (right image)

Image J (Version 1.51, https://imagej.nih.gov/ij/download/) with the plugin FracLac used to estimate FD. The plugin applies the boxcounting method to approximate FD. An ROI position was selected as described above and the settings “draw grid” and “regression” were ticked. This produces a plot of the regression line; the slope of which is then representing the FD value. The tool operates on the binarized image (Fig. 1) and approximates FD from:

1 $$ FD=\frac{\log N}{\log D} $$

with *N* representing the number of grid cells (boxes) and *D* denoting the scale of the grid.

Intra-observer reproducibility of the FD measurements was assessed by 6 repeated measurements (including definition of the ROI) in 10 individuals.

### Statistical analysis

Since a normal distribution by means of the Shapiro-Wilk-test could not be safely assumed for all FD values, FD was compared between the study and the control group using the Mann-Whitney *U* test. A 5% level of significance was assumed throughout the study. Influence of co-variables like age and sex was assessed by analysis of variance and Pearson correlation. Intra-observer reproducibility was measured by means of the intra-class coefficient (ICC) using a two-way mixed-effects model with absolute agreement, single rater/measurement as input [15, 16]. R language and environment for statistical computing (R Core Team. R (2017) A language and environment for statistical computing. Vienna, Austria; URL http://www.R-project.org/) was used for the statistical analysis (additional packages: ‘irr’ and ‘psych’). A posteriori power calculation was conducted using the R-library ‘pwr.’

## Results

When combining all FD data for the study and control group, normal distribution within these groups cannot safely be assumed (*p*_Shapiro-Wilk_: study = 0.0538, control = 0.02361).

**Fig. 2:**
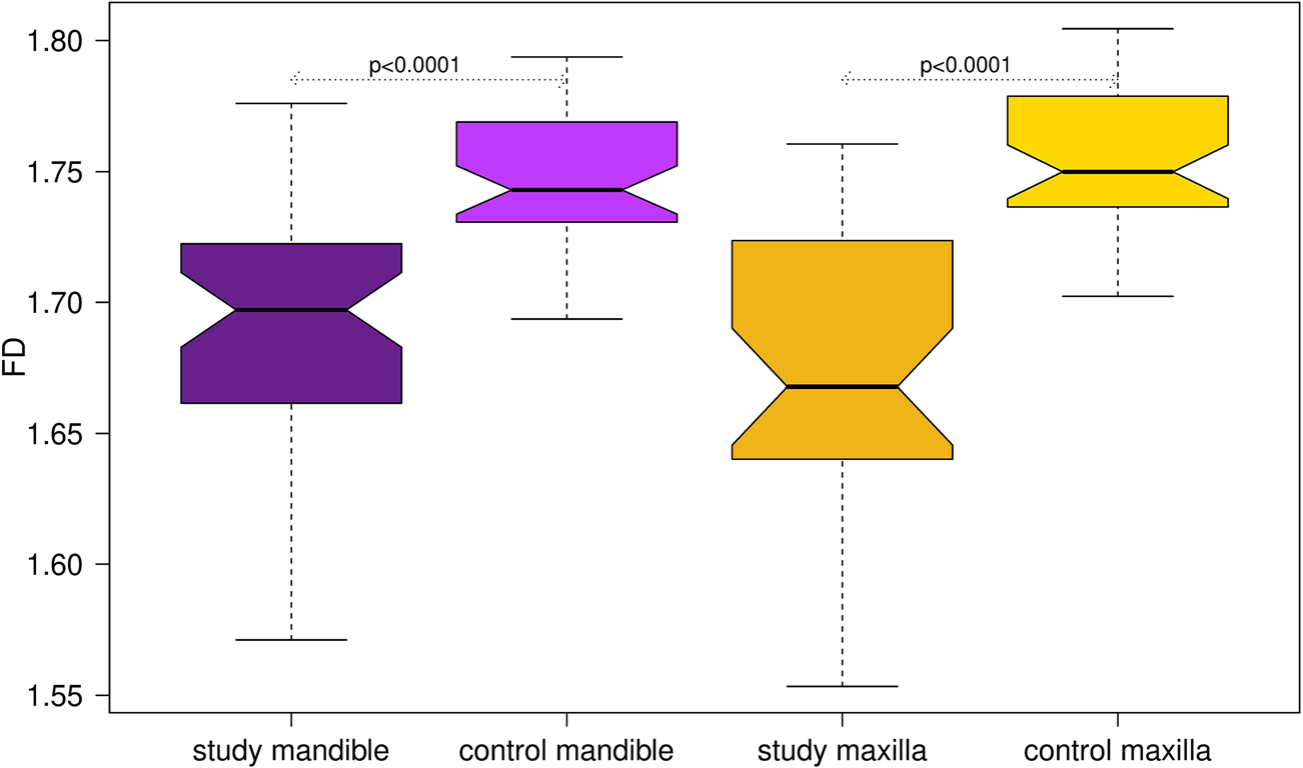
Boxplots of the FD values (fractal dimension) for study and control group for both jaws separately. The boxes range (interquartile range, IQR) from the upper to the lower quartile of the data. The whiskers denote those values at a distance of ± 1.5 IQR from the respective upper/lower box-boundary value

Mean age in the control group (57.6 ± 12.2 years) was significantly (*p* < 0.0001) lower than that in the study group (76.5 ± 9.4 years). Gender distribution was equal (male/female study group: 37/43, control group: 40/44). A summary of FD estimates is provided in Table 1. FD differed highly significantly (*p* < 0.001) between control and study group for both jaws (Fig. 2). When combining all FD values for each jaw, no difference was observed between mandibular and maxillary FD.

**Table 1:** Descriptive statistics of the fractal dimension values separated for both jaws. *SD* standard deviation (study: study group, control: control group)

	Maxilla		Mandible	
	Study	Control	Study	Control
Mean	1.674	1.755	1.691	1.747
SD	0.0565	0.0261	0.0459	0.0246
Median	1.668	1.750	1.697	1.743
Min	1.553	1.702	1.571	1.694
Max	1.761	1.804	1.776	1.794

Mean (± standard deviation) FD in the study group (1.684 ± 0.051) was roughly 3.5 % lower (*p*_Mann-Whitney-U_ < 0.0001) than that in the control group (1.745 ± 0.026).

**Fig. 3:**
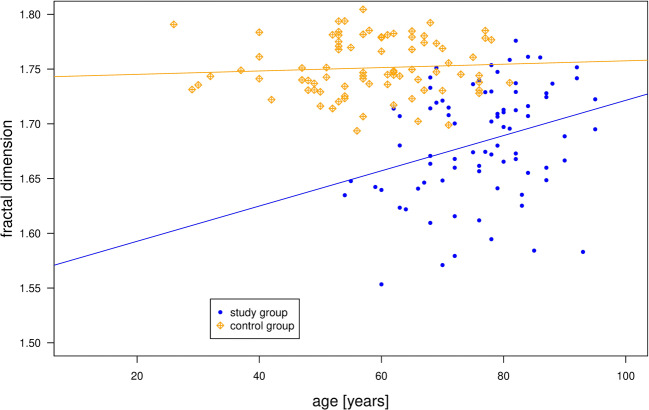
In both groups, FD values slightly increased with age, although this trend seems not to be well represented by a linear relationship in the study group (blue)

**Fig. 4:**
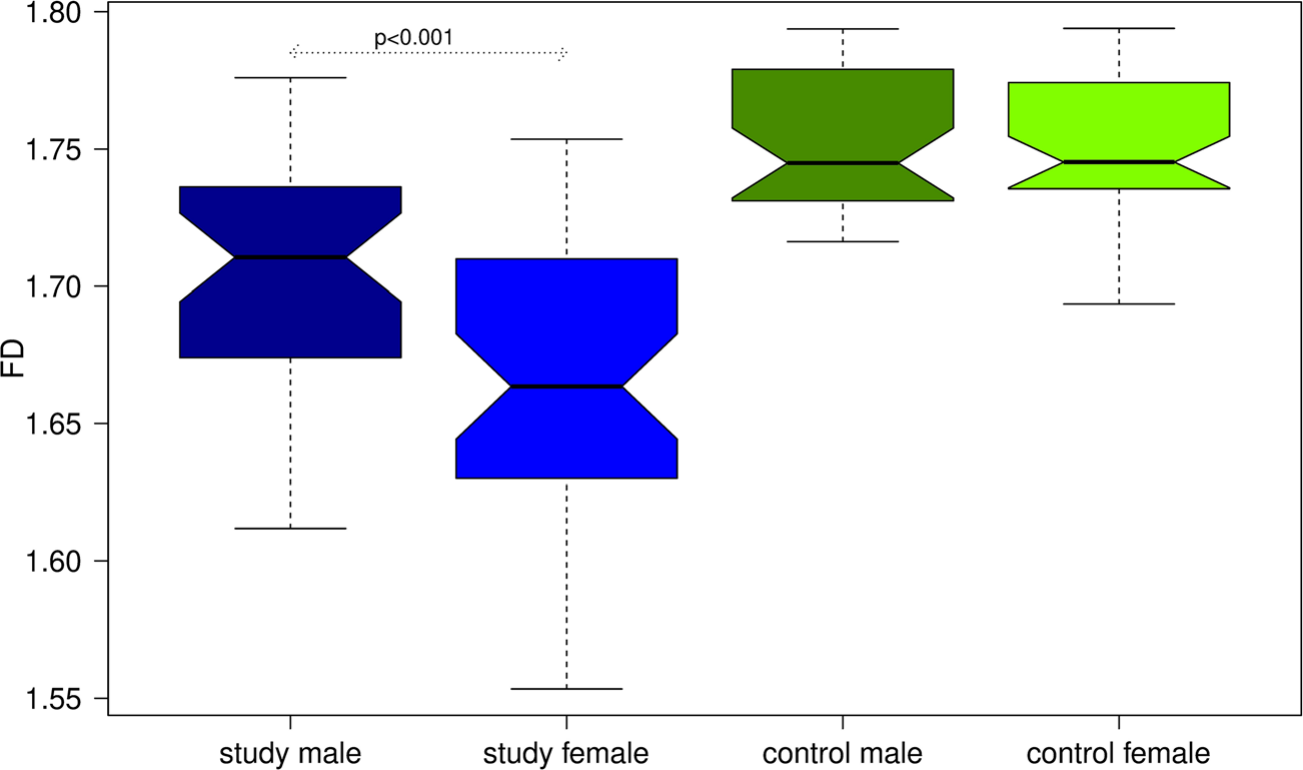
Boxplot of FD values separately for male/female individuals. For detailed explanation of the boxplots, please refer to Fig. 2

The paired Wilcoxon test revealed a significant (*p* < 0.001) influence of sex and age on FD values in the study group, yet not in the control group (Fig. 3). With increasing age, FD values within this group also increased (Spearman rho: 0.2895, *p* < 0.05). For the study group, FD values were slightly higher for males (Fig. 4).

**Fig. 5:**
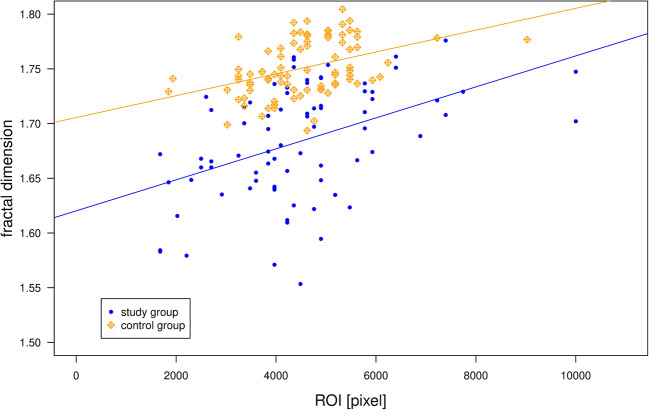
A clear positive correlation between FD value and ROI size can be observed for both study and control groups

The square ROI range resulting from repeated manual selection in this process ranged between 3249 and 6241 pixel (mean: 4752 pixel). ROI differences between study and control group were non-significant (*p*__Wilcoxon_: 0.338). FD correlated positively (*p* < 0.0001) with ROI size for both control and study group in both jaws (Fig. 5).

Reproducibility of the FD assessments was good (ICC: 0.87, 95% confidence interval: 0.72 to 0.96).

## Discussion

Antiresorptive medication such as bisphosphonates alters the spatial appearance of bone. Typical signs include osteolysis, dense woven bone, a thickened lamina dura, subperiosteal bone deposition, and persisting alveolar sockets after tooth extraction with lacking bone remodeling [6]. These characteristics render fractal analysis a potential tool for numerical evaluation. Our study aimed to compare fractal dimension values on a MRONJ study with a control. Records and CBCTs from both were taken from existing databases in our University Medical Center. As the measurements were conducted on two-dimensional (2D) ROIs extracted from CBCT scans, FD values in this study determine 2D complexity of bony architecture. FD values were estimated with the open-source tool FracLac from the Image J family. This approximates FD by means of the boxcounting method. Boxcounting refers to a process applying a grid of square boxes to a binary (black/white) image and subsequently counting the number of boxes (cells) that include the boundary structure (bone boundary). As the grid size decreases, the number of cells including the boundary increases. From this inverse relation, the fractal dimension is computed according to Eq. (1). In theory, for the typical radiographic signs in patients suffering MRONJ, one would expect that the bone structure is becoming coarser, i.e., less complex. This theory is supported by our findings exhibiting a highly significant FD decrease in the study group when compared with control. That this difference is also clinically relevant as illustrated in Fig. 2 since neither for the mandible nor the maxilla the boxes of study and control overlap. Interestingly, the authors in [14] observed a contrary trend of higher FD values in the study group. However, the difference was very small and only significant in one out of four ROIs [14]. According to the theoretical considerations explained before, one should expect lower FD values for MRONJ patients. Thus, our observations seem to be in agreement with the theory behind fractal dimension.

Intra-rater reproducibility was good in this study according to the criteria formulated in [15]. As only one observer (RB) assessed the images, however, we cannot conclude on inter-observer variance.

Our findings suggest that FD could potentially be used as a numeric discrimination measure to separate diseased from non-diseased individuals. Other studies should be encouraged to further look into this interesting relationship. While we did not find differences in FD values between the lower and upper jaw, interestingly we observed a significant decrease of FD with age in the study group. In this group, FD values were also significantly higher in male than in female (Fig. 4). This finding is similar to that reported for panoramic radiography [17]. However, Sindeaux and colleagues [18] also using panoramic radiography observed contradictory results for different regions of interest. Thus, this finding should be further investigated in studies particularly designed to investigate gender effects on fractal dimensions in jaw bones.

Several shortcomings are involved with our study. First of all, we failed to sample a control group from patients we had CBCT images available that was really similar in age to the study group. As age significantly influenced FD values in the study group with a weak to moderate positive correlation, this drawback may have influenced our results. However, due to the retrospective nature of our study design and availability of CBCTs from healthy patients in our database, unfortunately no other control group was available. Although correlation between FD and age was small in our study, future studies should aim to include an age- and gender-matched control group to avoid this effect. Post hoc power analysis modeled as one-way analysis of variance (ANOVA) tests revealed a power of 1.0 for our sample size (≧ 80 in each group). Although the normal distribution at least for the control group could not be assumed, the power of the study even under very conservative assumptions can be considered high.

It is also important to note that the selection of the ROIs according to anatomical criteria had been initially decided to produce comparable results to those reported in [14]. This selection process, however, did not differentiate between clinically necrotic bones and only altered due to the bisphosphonate medication. Thus, from our results we cannot conclude on potential FD differences between them. Apparently, our selected region was capable to capture bone that was already altered by the medication in such that the FD values were significantly lower than in a healthy population. It can be speculated that FD values in the group of necrotic bone should be even smaller than those in the non-necrotic group. Yet this needs to be further investigated in future studies.

Another interesting factor seems to be the ROI size used for FD estimation. We observed a highly significant positive correlation between FD values and number of pixels in the ROI (see Fig. 5). This finding can be explained by the fact that increasing the ROI results increases the number of scale factors *D*, i.e., more data points for FD estimation. Also, the number of boxes per scale is increased, thus resulting in a more stable computation of FD [19].

As we only had a 2D tool available for FD estimation (FracLac in combination with Image J), our analysis was restricted to a 2D analysis. However, the image data were inherently 3D; thus, a 3D analysis could probably provide even more accurate information. This could be an interesting option for a similar follow-up study, i.e., using 3D fractal dimension estimation also based on boxcounting. Apart from FD, it would be also interesting to investigate other bone morphological parameters for this disease.

Despite these shortcomings involved with our study, our results seem to indicate that fractal analysis using the boxcounting method provides a good discriminator between healthy individuals and those suffering from MRONJ. Sufficiently large ROI sizes should be aimed for to ensure stable and reproducible FD estimates. The additional influence of co-factors age and sex should be investigated in future investigations.
